# Nickel allergy is associated with a broad spectrum cytokine response

**DOI:** 10.1111/cod.14199

**Published:** 2022-09-08

**Authors:** Niels P. J. De Graaf, Sanne Roffel, Susan Gibbs, Cees J. Kleverlaan, Marta Lopez Gonzalez, Thomas Rustemeyer, Albert J. Feilzer, Hetty J. Bontkes

**Affiliations:** ^1^ Department of Dermatology, Amsterdam UMC Vrije Universiteit Amsterdam Amsterdam The Netherlands; ^2^ Department of Dental Materials Science, Academic Centre for Dentistry Amsterdam University of Amsterdam and Vrije Universiteit Amsterdam Amsterdam The Netherlands; ^3^ Department of Oral Cell Biology, Academic Centre for Dentistry Amsterdam University of Amsterdam and Vrije Universiteit Amsterdam Amsterdam The Netherlands; ^4^ MOVE Research Institute Amsterdam The Netherlands; ^5^ Department of Molecular Cell Biology and Immunology, Amsterdam UMC Vrije Universiteit Amsterdam Amsterdam The Netherlands; ^6^ Amsterdam Infection and Immunity Amsterdam The Netherlands; ^7^ Department of Medical Oncology Amsterdam UMC Amsterdam The Netherlands; ^8^ Cancer Center Amsterdam Amsterdam The Netherlands; ^9^ Department of Clinical Chemistry, Medical Immunology Laboratory Amsterdam UMC Amsterdam The Netherlands; ^10^ Amsterdam Gastroenterology Endocrinology Metabolism Amsterdam The Netherlands

**Keywords:** allergy, contact dermatitis, cytokines, IL‐5, nickel

## Abstract

**Background:**

Nickel‐induced proliferation or cytokine release by peripheral blood mononuclear cells may be used for in vitro diagnosis of nickel allergy.

**Objectives:**

Aim of this study was to explore the nickel‐specific cytokine profile to further elucidate the pathogenesis of nickel allergic contact dermatitis (ACD) and to identify potential new biomarkers for nickel ACD.

**Methods:**

Peripheral blood mononuclear cells from patients and controls were cultured with T‐cell skewing cytokine cocktails and/or nickel. Cytokine and chemokine concentrations were assessed in culture supernatants using validated multiplex assays. Specific cytokine production was related to history of nickel allergy and patch‐test results.

**Results:**

Twenty‐one of the 33 analytes included in the analysis were associated with nickel allergy and included type1 (TNF‐α, IFN‐γ, TNF‐β), type 2 (IL‐3, IL‐4, IL‐5, IL‐13), type 1/2 (IL‐2, IL‐10), type 9 (IL‐9), type 17/1 (IL‐17A[F], GM‐CSF, IL‐21) and type 22 (IL‐22) derived cytokines as well as the T‐cell/antigen presentation cell derived factors Thymus and activation regulated chemokine (TARC), IL‐27 and IP‐10. Receiver operator characteristics (ROC) analysis showed that IL‐5 was the strongest biomarker for nickel allergy.

**Conclusions:**

A broad spectrum of 33 cytokines and chemokines is involved in the allergen‐specific immune response in nickel allergic patients. IL‐5 remains, next to the lymphocyte proliferation test, the strongest biomarker for nickel allergy.

## Graphical abstract

- PBMC from nickel allergic patients and controls were stimulated with nickel under type 1, 2 and 17 skewing culture conditions; levels of 33 cyto‐ /chemokines were analysed and related to medical history of nickel contact dermatitis and nickel patch test result.
- A broad spectrum of 21 type 1, 2, 9,17, 22 and antigen presenting cell related factors was associated with nickel allergy.
- IL‐5 production under type 2 skewing conditions and lymphocyte proliferation are most potent biomarkers for nickel allergy.

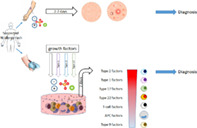

## 1 INTRODUCTION

Nickel is the best known and most frequent of the metals causing allergic contact dermatitis (ACD).^1^ Nickel can be found in jewellery, medical implants, dental devised, cooking pans and items that are generally used. Nickel allergy is a type‐IV hypersensitivity reaction, upon interaction with antigen presenting cells nickel‐specific CD4^+^ T‐cells are primed and activated.^2^ Nickel is also capable of directly activating dendritic cells via Toll‐like receptor‐4, thereby inducing inflammatory signalling via NF‐κB.^3^ The gold standard in diagnosing nickel contact allergy is the patch test.^4^ However, this test is particularly suited for ACD, not for complaints due to internal exposure from medical implants and oral exposure from dental devices. Irritant reactions my cause false positive results, while insufficient allergen skin penetration may lead to a false negative result. Furthermore, there is a small risk of patient sensitization.^5^

As we described previously, an optimized lymphocyte proliferation test (LPT) using CFSE can also be considered as a good diagnostic tool for nickel allergy.^6^ In vitro cytokine production in response to nickel has also been shown to differentiate between nickel allergic and non‐allergic patients. Pro‐inflammatory cytokines such as IFN‐γ, IL‐2, Il‐12, IL‐4, IL‐5, IL‐8 and regulatory cytokines such as IL‐10 and TGF‐β1 have been investigated.^7^, ^8^, ^9^, ^10^, ^11^ Production of ‘type 2’ cytokines IL‐2, IL‐13 and IL‐5, showed the best correlation with contact allergy to nickel.^10^ However, studies investigating the role of inflammatory mediators in nickel allergy have been limited to a few selected cytokines and chemokines, and thus only provide information on restricted profiles. An evaluation of the broader inflammatory profile of nickel specific T‐cells may provide more insight into the pathogenesis and immunological pathways of metal allergy and may help in the development of improved diagnostic tests for metals such as titanium, for which patch tests proved to be unreliable.

Using multiplex assays, a broad range of inflammatory mediators can be determined in a small volume sample. In this study, peripheral blood mononuclear cells (PBMC) of in total 52 nickel allergic patients and controls were stimulated in vitro with nickel sulfate (NiSO_4_). To study whether sensitivity of the cyto‐/chemokine release assay can be enhanced, cytokine cocktails skewing lymphocytes towards type‐1, ‐2 or ‐17 function were added. A wide range of 33 different chemokines, cytokines and growth factors was analysed, most of which, to our knowledge, have not previously been examined in an in vitro assay in response to nickel. The aim of this study was to identify novel cytokines in the inflammatory mediator profile induced by nickel and to investigate whether existing proliferation and type2 cytokine production diagnostic tests can be improved.

## 2 METHODS

### 2.1 Patients

The study followed the Declaration of Helsinki Principles. All participants gave written informed consent, and the study was approved by the medical ethical committee of VU University Medical Centre (Central Committee on Research Involving Human Subjects protocol number: NL52668.029.15). Patient inclusion was as described previously.^6^ In short, 52 participants who presented at our out patients clinic for evaluation of suspected cutaneous hypersensitivity were patch tested with NiSO_4_ petrolatum and included in the study; 27 with a positive patch test and 25 with a negative patch test to nickel. Information on history of AVD to nickel containing metal alloys, based on patients answers to questions regarding the development of skin rash after contact with (metal) jewellery, coins, metal tools, scissors or metals in clothing such as belts and buttons, was recorded, enabling us to divide the participants into four groups: true positives (positive patch test and history), true negatives (negative patch test and history) and two groups in which the patch test did not confirm the history. The subjects were without systemic immunosuppression or UV radiation therapy. Patient characteristics have been previously described, (6) and are listed in Table S1. All participants donated peripheral blood samples (heparinized), which was used for nickel sensitization testing using the lymphocyte cytokine production test.

### 2.2 Patch tests

Patch testing was performed according to the guidelines of the International Contact Dermatitis Research Group (ICDRG) criteria. All included patients were tested with at least the 30 allergens of the European baseline contact allergen series, on their back with an application time of 48 h. Allergens were purchased from Van der Bend (Brielle, The Netherlands). The patch test concentration of NiSO_4_ was 5% in petrolatum as recommended in the European baseline test Series. Allergens were tested in square chambers (Van der Bend, Brielle, the Netherlands) mounted on Fixomull stretch from Beiersdorf (Hamburg, Germany).

Readings were performed at 48, 72 and 168 h (7 days). Positive reactions were rated as +, ++ or +++.

### 2.3 Lymphocyte cytokine production test

The lymphocyte cytokine production test (LCPT) was performed in the laboratory of the Oral Cell Biology department of the Academic Centre for Dentistry Amsterdam (ACTA), cultures were set up as previously described.^6^ Supernatants of these cultures were collected for multiplex analysis. Briefly, peripheral venous blood (40 ml) samples (heparinized) were collected and then peripheral blood mononuclear cells (PBMCs) were isolated on Ficoll Histopaque (Sigma‐Aldrich Chemie GmbH, Taufkirchen, Germany). After isolation, the cells were cultured in medium (IMDM, 100 IE/ml Na‐penicillin and 100 μg/ml streptomycin, 2 mM l‐glutamine [Biochrom Seromed, Berlin, Germany] and 50 μM β‐mercapto‐ethanol) containing 10% autologous serum. The final cell density was 7.5 × 10^5^ cells/ml/well. The PBMC were cultivated with or without 50 μM NiSO_4_ (Merck), the same salt as used in the patch test, for 7 days in duplicate. We have previously shown 50 μM NiSO_4_ to be the optimal non‐toxic concentration.^9^, ^10^ In order to increase allergen‐induced T‐cell cytokine production, growth factor supplements consisting of IL‐7 (0.1 ng/ml; Strathmann Biotec AG, Hamburg, Germany), IL‐12 (1 ng/ml; PeproTech, NJ, USA), IL‐4 (3 ng/ml), IL‐1β (10 ng/ml) and IL‐23 (10 ng/ml), all from R&D systems (Minneapolis, MN, USA), were used during culture as described previously.^8^ The combination of IL‐7, and either IL‐12, IL‐4 or IL‐1β/IL‐23 were added to respectively, to skew towards the release of type 1, type 2 or type 17 cytokines. As a positive control, the T‐cell mitogen phytohaemagglutinin (PHA; Merck) (3 μg/ml) was used. Negative controls were defined as unstimulated cells. On day 7, culture supernatants were harvested and stored at −30°C until multiplex analysis.

### 2.4 Multiplex analysis

Cytokine production was determined by collecting supernatants at Day 7 of culture. The analysis of mediators in the supernatants was performed using the mesoscale platform and V‐PLEX validated immunoassays from MSD (Rockville, MD). The following kits were used: Cytokine panels 1 and 2, Pro‐inflammatory panel 1, Chemokine panel 1 and Th17 panel. See for details on individual analytes in these kits and analytes excluded from analysis Table S2. The results of the cytokine production test are presented as the stimulation index (SI), which is calculated by the cyto‐/chemokine production (pg/ml) in the nickel stimulated cultures divided by the production in unstimulated cultures. To avoid disproportionately high stimulation indexes, a lower cut‐off of 1 pg/ml was used.

### 2.5 Statistical analysis

SPSS (version 18.0.2, IBM SPSS Inc., SPAW Statistics, New York, NY, USA), MedCalc (version 11.6.1.0, MedCalc Software bvba, Mariakerke, Belgium) and GraphPad Prism (version 7.00, GraphPhad Software, La Jolla, CA, USA) were used for statistical analyses. Supervised and unsupervised clustering and heatmap generation was performed using R. To better interpret the heat map cluster results, the top 10% of the SI's were lowered, resulting in a top SI of 44.2. To determine optimal SI cut‐off values to distinguish positive from negative patients, receiver operator characteristics (ROC)‐curve analyses were performed for each in vitro parameter. Statistical significance was defined as *p* < 0.05.

## 3 RESULTS

A summary of the patient characteristics are listed in Table S1. Of the 52 included patients 15 were defined as ‘true negatives’ because they had no clinical history of nickel allergy and were patch test negative, this is the control group. Twenty patients were defined as “true positives” because they had a clinical history of nickel allergy and were nickel patch test positive (nickel allergic). The remaining patients had either a clinical history of nickel allergy with a negative patch test (PT − H+, *n* = 10) or a positive patch test without a clinical history of nickel allergy (PT + H−, *n* = 7).

Five different optimized V‐plex kits were used to analyse 44 analytes. Eight of these were excluded from analysis because baseline production (medium) exceeded the upper limit of the standard curve and thus the upper limit of detection of the assay, these were predominantly chemokines (see Table 2). IL‐7 was excluded because it was added during culture. IL‐17D was excluded because it is not produced by PBMC, and was undetectable in all cultures. IL‐8 was analysed with two different assays, the sensitive assay results were excluded (all values above upper limit of detection of the assay), but results with the assay suited for high levels (IL‐8 HA) were included.

IL‐15, IL‐17C, TSLP, IL‐23 and IL‐31 were not detectable in most patients after stimulation with Nickel. IL‐15 and IL‐31 were produced after stimulation with PHA, while IL‐17C, TSLP and IL‐23 production was also not induced by PHA stimulation (not shown).

IL‐1α, VEGF‐A, IL‐16, IL‐1β, IL‐12p70, Eotaxin‐1 and Eotaxin‐2, were produced but the mean SI was <1.5 in all culture conditions.

**FIGURE 1:**
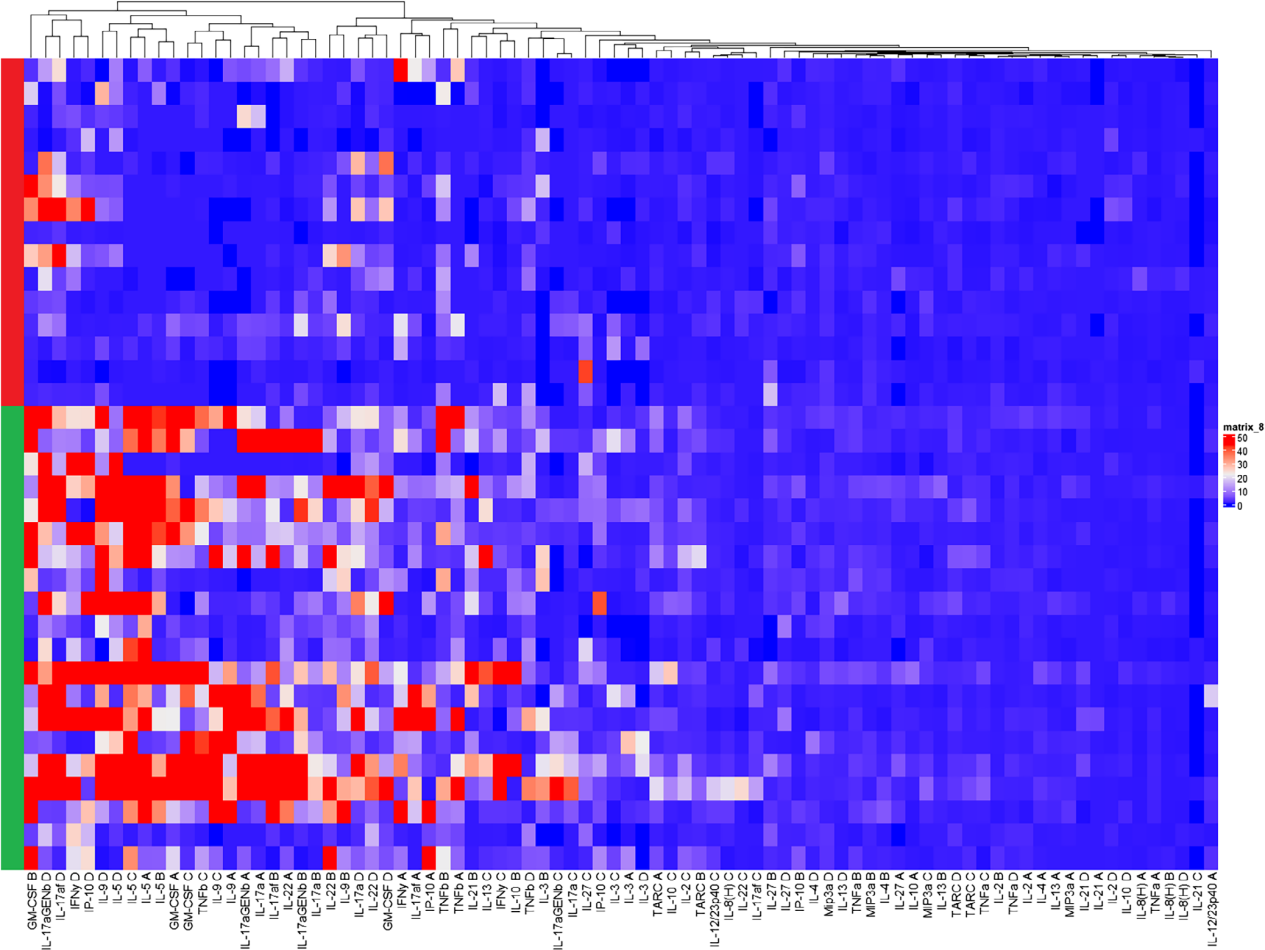
Supervised cluster analysis: Dark blue colours represent low Stimulation Index (SI)'s, white colours represent average SI's, and dark red represent high SI's. Controls (patch test negative, no history of nickel allergy: 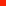 true positives (patch test positive, history of nickel allergy: 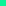 A, non‐skewed; B, type 1 skewed; C, type 2 skewed; D, type 17 skewed cultures.

All remaining analytes were included in the supervised cluster analysis shown in Figure 1, with the exception of IL‐4 production in the type2 skewing culture condition as IL‐4 was added to the culture medium, and the IFN‐γ production in the type 1 skewing condition due to high background production. IL‐17A was analysed with two different assays; IL‐17A and IL‐17A genB which had different detection ranges (0.74–3633 and 0.413–1950 pg/ml, respectively), both assays were included in the analysis (see also Table S2 for details on included and excluded analytes).

**FIGURE 2:**
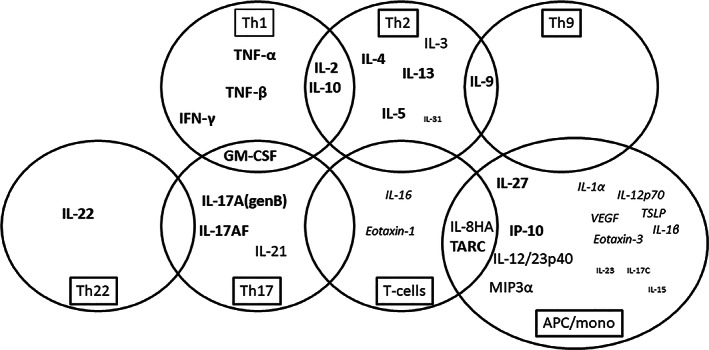
Analytes included in the analysis. Large bold font: showed a good to excellent accuracy for diagnosis (area under the curve [AUC] ≥ 0.80) in one or more of the culture conditions. Large font: showed a poor to fair accuracy for diagnosis (AUC 60–80) in one or more of the culture conditions. Medium font italic: detectable but Stimulation Index (SI) <1.5 in all patients. Small font: not detectable in more than 50% of controls and patients.

The supervised cluster analysis in Figure 1 shows that a broad spectrum of cytokines is associated with nickel allergy, these cytokines include type 1, type 2, type 9, type 22 and type 17 derived cytokines, demonstrating that the response to nickel is not restricted to one T‐cell subset. Unsupervised cluster analysis also shows clustering of controls (true negatives) and patients with both a positive patch test and a history of nickel allergy (true positives). The two intermediate groups having either a history nickel allergy but a negative patch test or no history of nickel allergy with a positive patch test do not cluster preferentially with either the controls of true positives (Figure S1). Figure 2 summarizes the variety in cytokines associated with nickel allergy, a type 2 response seems to dominate, however. ROC analysis using the true negative patients as controls and true positive patients as cases, showed that IL‐5 production both under no skewing and type 2 skewing conditions and even under type 1 skewing conditions have the best accuracy of the top 10 analyte/culture condition combinations with an area under the curve of ≥0.90 (Table 1). Furthermore, other cytokines in this top 10 are the type 2 associated cytokine IL‐13, IL‐2 produced under type 2 skewing conditions and Thymus and activation regulated chemokine (TARC), which predominantly attracts CCR4 positive Th2 cells. In addition, IL‐22 (under no skewing and type 17 skewing culture conditions) produced by type 22 cells and associated with type 17 responses, GM‐CSF (produced by type 1/type 17 cells) and TNF‐β (under type 2 skewing conditions) produced by type 1 cells show a strong correlation with nickel allergy.

**TABLE 1:** Analytes with an excellent AUC value (≥0.90)

Analyte	Condition	Th source	ROC cut off	AUC	95% CI	*p*‐Value ROC	Sens	95% CI	spec	95% CI	Accuracy	95% CI
IL‐5	Type 2	Th2	>2.5	0.94	0.86–1.0	<0.0001	90	68.3–98.8	100	78.2–100	94.3	80.8–99.3
IL‐5	No skewing	Th2	>3.0	0.95	0.88–1.0	<0.0001	85	62.1–96.8	93.3	68.1–99.8	88.6	73.3–96.8
IL‐5	Type 1	Th2	>1.8	0.90	0.78–1.0	<0.0001	85	62.1–96.8	93.3	68.1–99.8	88.6	73.3–96.8
IL‐13	Type 2	Th2	>1.6	0.90	0.79–1.0	<0.0001	80	56.3–94.3	100	78.2–100	88.6	73.3–96.8
TNFβ	Type 2	Th1	>2.5	0.90	0.80–1.0	<0.0001	85	62.1–96.8	93.3	68.1–99.8	88.6	73.3–96.8
GM‐CSF	No skewing	Th1/17	>2.7	0.93	0.85–1.0	<0.0001	85	62.1–96.8	86.7	59.5–98.3	85.7	69.7–95.2
IL‐22	No skewing	Th22	>3.7	0.93	0.84–1.0	<0.0001	75	50.9–91.3	93.3	68.1–99.8	82.9	66.4–93.4
IL‐22	Type 17	Th22	>4.6	0.93	0.85–1.0	<0.0001	85	62.1–96.8	80.0	91.9–95.7	82.9	66.4–93.4
TARC	No skewing	T/APC	>1.7	0.90	0.80–1.0	<0.0001	75	50.9–91.3	93.3	68.1–99.8	82.9	66.4–93.4
IL‐2	Type 2	Th1/2	>2.5	0.90	0.80–1.0	<0.0001	60	36.1–80.9	93.3	68.1–99.8	74.3	56.7–87.5
IL‐5/IL‐8 SI	No skewing		>3.8	0.95	0.88–1.0	<0.0001	85	62.1–96.8	93.3	68.1–99.8	88.6	73.3–96.8

Abbreviations: AUC, area under the curve; CI, confidence interval; ROC, receiver operator curve; sens, sensitivity; SI, stimulation index; spec, specificity.

In Figure 2, other cytokines that are associated with nickel allergy with a good‐excellent accuracy (AUC ≥0.80) in one or more culture conditions are shown in bold, which include TNF‐α, IFN‐γ, IL‐10, IL‐4, IL‐17A, IL‐17AF and IL‐27 and IP‐10. For more information on AUC and *p*‐values for the different culture conditions, see Table S3. IL‐27 and IP‐10 are antigen presenting cell (APC) derived factors which may be indirectly induced by specific T‐cells under type17 and type 2 skewing conditions respectively (Table S3).

**FIGURE 3:**
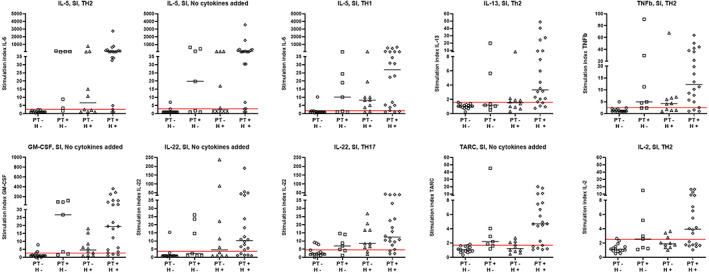
Stimulation Index (SI) of all patient groups of the analyte and culture conditions which showed an area under the curve [AUC] ≥ 0.90 in the receiver operator curve (ROC) analysis based on controls and ‘true positives’. Red line represents the cut off based on ROC analysis. H, history of nickel allergy; PT, patch test

Figure 3 shows the results for the cytokine production culture condition combinations that had an excellent accuracy for diagnosis (AUC ≥ 0.90). Responses in all groups are shown, including patients without a nickel allergy in their clinical history with a positive patch test (PT + H−) and patients with a nickel allergy history but negative in the patch test (PT − H+). For most of the cytokines positive responses are detected in both these groups. Table S4 shows the actual production in pg/ml for medium control and nickel stimulated cultures.

We have previously shown in this patient group that specific proliferation to nickel is associated with nickel allergy. We therefore analysed the correlation between the LPT and the LCPT for the cytokines with an AUC ≥ 0.90 under non‐skewed and type 2 skewed conditions. Indeed, the LPT and cytokine production under non‐skewed conditions (IL‐5, GM‐CSF, IL‐22, TARC production) and type 2 skewing conditions (IL‐5, IL‐13, IL‐2, TNF‐β production) all correlated well (*p* < 0.0001, see Table S5).

In addition, we calculated the percentage of patients and controls that are positive or negative in both the LPT and the four best performing cytokines under non skewing conditions (Table 2). Subsequently, we calculated the sensitivity and specificity for the whole patient group, using nickel allergy in the medical history as reference (Table 3). Sensitivity and specificity of the patch test are then 67% and 68%, respectively. These are increased when IL‐5 production is analysed under no skewing conditions (73% and 77%) or type 2 skewing conditions (80% and 73%). We previously demonstrated that the LPT under non‐skewing conditions showed an even higher sensitivity (83%) and specificity (86%), demonstrating that the LPT is a good in vitro test to diagnose nickel allergy. A combination of the proliferation test and IL‐5 production test increases the specificity (91%) with some loss in sensitivity (70%).

**TABLE 2:** results from LPT and LCPT combined under non‐skewed culture conditions.

PT/History	Double negative (%)	Double positive (%)
IL‐5		
+/+ (*n* = 20)	5	85
−/+ (*n* = 10)	30	40
+/− (*n* = 7)	43	29
−/− (*n* = 15)	87	0
GM‐CSF		
+/+ (*n* = 20)	5	85
−/+ (*n* = 10)	50%	20
+/− (*n* = 7)	29	29
−/− (*n* = 15)	73	0
IL‐22		
+/+ (*n* = 20)	5	85
−/+ (*n* = 10)	20	40
+/− (*n* = 7)	43	29
−/− (*n* = 15)	80	0
TARC		
+/+ (*n* = 20)	5	70
−/+ (*n* = 10)	30	30
+/− (*n* = 7)	43	29
−/− (*n* = 15)	87	0

Abbreviations: double negative, negative in the LPT and in the LCPT; double positive, positive in both LPT and LCPT assay; LCPT, Lymphocyte cytokine production test; LPT, lymphocyte proliferation test; PT, patch test.

**TABLE 3:** Characteristics of LPT and IL‐5 LCPT in the whole patient group, history of nickel allergy is reference

Test	Condition	Sens	95% CI	Spec	95% CI	Accuracy	95% CI
PT	Not applicable	67	47.2–82.7	68	45.1–86.1	67.3	52.9–79.7
IL‐5 LCPT	Type 2 skewing	80	51.4–92.3	73	49.8–89.3	76.9	63.2–87.5
IL‐5 LCPT	No skewing	73	54.1–87.7	77	54.6–92.2	75.0	61.1–86.0
LPT	No skewing	83	65.3–94.4	86	65.1–97.1	84.6	71.9–93.1
LPT and IL‐5 LCPT	No skewing	70	50.6–85.3	91	70.8–98.9	79.0	65.3–88.9

Abbreviations: CI, confidence interval; LCPT, lymphocyte cytokine production test; LPT, lymphocyte proliferation test; PT, patch test; Sens, sensitivity; Spec, specificity.

## 4 DISCUSSION

Specific IL‐2 production in response to nickel in allergic patients has been shown more than 30 years ago.^12^ In subsequent studies by us and others specific TNF‐α, IFN‐γ, IL‐2, IL‐4, IL‐5, IL‐10, IL‐13 and IL‐17 production in relation to nickel allergy upon in vitro stimulation of PBMC has been demonstrated.^7^, ^8^, ^10^, ^11^, ^13^, ^14^, ^15^, ^16^, ^17^, ^18^ Some of these studies could not confirm specific production of one or more of these cytokines (TNF‐α, IFN‐γ, IL‐4, IL‐10 or IL‐17), possibly due to small group sizes or low sensitivity assays.^11^, ^12^, ^13^, ^14^

In this study, using a sensitive analysis platform, we confirm specific production of aforementioned cytokines and 13 additional factors (21 of the 33 cyto‐/chemokines analysed were produced specifically in response to nickel in nickel allergic donors), demonstrating that the T‐cell response to nickel shows a broad spectrum of type 1 (TNF‐α, IFN‐γ, TNF‐β), type 2 (IL‐3, IL‐4, IL‐5, IL‐13), type 1/2 (IL‐2, IL‐10), type 9 (, IL‐9), type 17/1 (IL‐17A[F], GM‐CSF, IL‐21) and type 22 (IL‐22) derived cytokines. The types 9 and 22 cytokines in particular are to our knowledge not previously described in this context. In addition, we show some cyto‐/chemokines (TARC, IL‐8, IL‐12/23p40, IL‐27 and IP‐10) which are predominantly produced by antigen presenting cells rather than T‐cells, to be associated with nickel allergy.

Of the tested T‐cell derived factors only IL‐16, Eotaxin‐1 and IL‐31 were not associated with nickel allergy. IL‐16 is produced by a variety of haematopoietic (a.o., CD8^+^ T‐cells, CD4^+^ T‐cells) cells and non‐haematopoietic (a.o., epithelial cells) cells and is a chemoattractant for CD4^+^ cells.^19^ It was shown to be specifically increased in response to nickel, in tape stripped skin samples of positive patch tests.^20^ Since we could not show specific production in our PBMC cultures, it is likely that IL‐16 is produced by keratinocytes of allergic individuals upon allergen contact and may be involved in the chemoattraction of (nickel responsive) CD4^+^ T‐cells.

IL‐31 belongs to the IL‐6 family and is predominantly produced by type 2 cells, skin homing CLA^+^ T‐cells and mast cells and functions via the IL‐31 receptor expressed on a.o. activated macrophages, eosinophils and keratinocytes.^21^ Despite being a type2 cytokine, IL‐31 was hardly or not detectable in our cultures, <0.5 pg/ml in the experimental conditions and 4–6 pg/ml in PHA stimulated control cultures. Not much is known about the role of Eotaxin‐1 in allergic contact allergy, although increased expression in skin has been shown after repeated irritation by sodium lauryl sulphate,^22^ and may therefore be more important for irritation rather than allergic contact allergy.

APC derived factors IL‐12/23p40 (type 2 skewing condition; AUC 0.78,), IL‐27 (type 17 skewing condition; AUC 0.85) and IP‐10 (particularly under type 2 skewing condition; AUC 0.87) were also associated with nickel allergy (Table S3). IL‐12/23p40 and IL‐27 are induced in healthy donor dendritic cells upon nickel stimulation while IFN‐γ can further boost production of these cytokines by antigen presenting cells.^23^, ^24^ The production of IP‐10 by antigen presenting cells is also predominantly induced by IFN‐γ.^25^ As nickel allergy associated IFN‐γ production was observed under all culture conditions (except type 1 skewing) it is likely that these associations are related to indirect activation of APC by cytokines produced by nickel specific T‐cells.

Expression of IFN‐γ, IL‐17A, IL‐22 expressing T‐cells were shown in biopsies of nickel inflamed skin by flow cytometry and immunohistochemistry, confirming the role of these cytokines in nickel ACD, however, type 2 cytokines were not analysed in these studies.^15^, ^26^ Liu et al. have shown increased expression of IFN‐γ, IL‐4, IL‐17A and IL‐9 by qPCR in nickel inflamed skin as compared to paired healthy skin.^27^ In skin biopsies of patients with chronic exposure to nickel, TNF‐α, IFN‐γ, IL‐4, IL‐13, IL‐17A and IL‐10 were detected by immunohistochemistry, in challenged skin IL‐2, IL‐23 and IL‐10 were shown to be expressed, however, no controls were included in this study.^28^ Overall, these data on local cytokine production upon nickel exposure confirm our data showing a role for a broad spectrum of type 1, type 2, type 9, TH17 and type 22 responses in nickel ACD.

The second aim of this study was to identify a cytokine (profile) after in vitro PBMC stimulation, which can most accurately predict nickel allergy and could be used as a diagnostic test. In the ROC analyses, there were 7 cytokines with an AUC of more than 0.90 with a *p* < 0.0001 (Table 1), of which the type 2 cytokines IL‐5 and IL‐13 are most accurate tests. Thus, even though we show here a broad spectrum of cytokines produced in response to nickel in nickel allergic individuals, the previously described in vitro IL‐5 production in response to nickel stimulation remains the best biomarker for nickel ACD and may be relevant for other metals.

Summer et al. described a lower production of IL‐8 in nickel allergic patients as compared to controls and reported IL‐5/IL‐8 ratio as the strongest diagnostic parameter for nickel allergy.^11^ A ratio of the IL‐5 SI/IL‐8 SI does not lead to an higher accuracy in our study as compared to the SI of IL‐5 alone (Table 1). Summer et al. did not correct for cytokine production in medium only cultures which may explain the discrepancy. As there is considerable variation in background production of the various cytokines measured in the medium control conditions (see also Table S4), it is in our opinion necessary to correct for background cytokine production.

Our previously described LPT and the IL‐5 LCPT are both more accurate than the patch test in this patient group, if nickel allergic contact history is used as a reference.^6^ The LPT without skewing is the most accurate test (85%), followed by the IL‐5 LCPT under type 2 skewing conditions (77%). The best choice may depend on the expertise and facilities available at a diagnostic laboratory. For the LPT, expertise in flowcytometry is needed, the proliferation can be assessed immediately after 6 days culture and results are available that same day. For the LCPT, it may be more cost effective to collect supernatants of multiple patients over time and analyse the supernatants for IL‐5 at a later stage if ELISA or a similar platform is used; this would lead to an increase in processing time. However, if a random access analyser is available to measure IL‐5 concentration in culture supernatants, this would be more cost effective than the LPT with a similar processing time, as less hands‐on time is needed. For a high specificity, the LPT and IL‐5 LCPT (without skewing) can be combined, but this leads to lower sensitivity and higher costs.

In conclusion, nickel allergic contact allergy is characterized by a broad cytokine response with cytokines related to type 1, type 2, type 9, type 17 and type 22 cells; type 2 cytokines showed the strongest correlation. The type 2 cytokine IL‐5 is the best biomarker for nickel allergy.

## AUTHOR CONTRIBUTIONS

*Investigation*: de Graaf NPJ, Rustemeyer T, Roffel S; Formal Analysis: de Graaf NPJ, Roffel S, Gibbs S, Lopez Gonzalez M and Bontkes HJ; Conceptualization: Feilzer AJ, Gibbs, S., Kleverlaan CJ, Bontkes HJ; Funding Acquisition and Supervision: Feilzer AJ, Gibbs, S. Writing‐Original Draft Preparation: Bontkes HJ, de Graaf NPJ; Writing‐review & editing: all authors.

## CONFLICT OF INTEREST

None of the authors have a conflict of interest.

## ETHICAL STATEMENT

The study was approved by the medical ethical committee of VU University Medical Centre, Central Committee on Research Involving Human Subjects protocol number: NL52668.029.15.

## Supporting information


**Table S1** patient characteristics
**Table S2**. MSD V‐plex kits used for mesoscale analysis
**Table S3**. Receiver operator characteristics (ROC) data, part 1
**Table S3**. Receiver operator characteristics (ROC) data, part 2
**Table S4**. Raw data (pg/ml) of top 10 analyte/culture condtition combinations
**Table S5**. Correlation lymphocyte proliferation and cytokine production tests

**Figure S1** Unsupervised cluster analysis: Dark blue colours represent low SI's, white colours represent average SI's, and dark red represent high SI's.

## Data Availability

Raw data are provided in the suplemental material.
