# Supplementary material for: Nickel allergy is associated with a broad spectrum cytokine response
Source: Contact Dermatitis. 2022 Sep 8;88(1):10–7. doi: 10.1111/cod.14199 (PMC10087880; doi:10.1111/cod.14199)
Supplement: Supplementary file 1 — Table S1 patient characteristics Table S2. MSD V‐plex kits used for mesoscale analysis Table S3. Receiver operator characteristics (ROC) data, part 1 Table S3. Receiver operator characteristics (ROC) data, part 2 Table S4. Raw data (pg/ml) of top 10 analyte/culture condtition combinations Table S5. Correlation lymphocyte proliferation and cytokine production tests [file COD-88-10-s002.docx]

**Supplemental Table 1: patient characteristics**

| **Characteristic** | **Controls** | **Nickel allergic patients** | **Patch test negative**  **History positive** | **Patch test positive**  **History negative** |
| --- | --- | --- | --- | --- |
| Age (range) | 61 (40-78) | 54 (25-75) | 60 (28-71) | 55 (30-81) |
| n (sex) | 9 (F), 6 (M) | 20 (F), 0 (M) | 9 (F), 1 (M) | 5 (F), 2 (M) |
| Patch test result | negative | 11 (+), 9 (++/+++) | negative | 5 (+), 2 (++/+++) |
| Mean time since patch test in months (SD) | 13.3 (15.2) | 11 (11.8) | 21 (33.4) | 12 (5.4) |

**Supplemental Table 2: MSD V-plex kits used for mesoscale analysis**

| **Cytokine**  **panel 1** | **Pro-inflammatory panel 1** | | **Chemokine**  **panel** | **Cytokine**  **panel 2** | **Th17**  **Panel** |
| --- | --- | --- | --- | --- | --- |
| GM-CSF† | IFNγ† | | IP-10† | IL-17A/F† | IL-17AgenB†^,2^ |
| IL-17A†^,2^ | IL-10† | | IL-8 HA†^,1^ | IL-3† | IL-21† |
| IL-5† | IL-13† | | TARC† | IL-9† | IL-22† |
| TNFβ† | IL-2† | |  |  | IL-27† |
| IL-12/23 p40† | IL-4† | |  |  | MIP-3α† |
|  | TNFα† | |  |  |  |
|  |  | |  |  |  |
| IL-1α^‡^ | IL-1β^‡^ | | Eotaxin-1^‡^ |  |  |
| VEGF-A^‡^ | IL12p70^‡^ | | Eotaxin-3^‡^ |  |  |
| IL-16^‡^ |  | |  |  |  |
|  |  | |  |  |  |
| IL-15^$^ |  | |  | IL-17C^$^ | IL-23^$^ |
|  |  | |  | TSLP^$^ | IL-31^$^ |
|  | |  |  |  |  |
| IL-7**^¶^** | IL-6**^¶^** | | MDC**^¶^** | IL-1RA**^¶^** |  |
|  | IL-8**^¶^**^,1^ | | MCP-4**^¶^** | IL-17D^††^ |  |
|  |  | | MIP-1α**^¶^** |  |  |
|  |  | | MIP-1β**^¶^** |  |  |
|  |  | | MCP-1**^¶^** |  |  |
|  |  | |  |  |  |
|  |  | |  |  |  |

†Analytes are included in heatmap analysis, with exception of IFNγ production in the Type 1 skewing condition due to high background and IL-4 production in the Type 2 skewing condition because IL-4 was added during culture.

^‡^Analytes excluded from heatmap analysis because mean SI in the patient group were all <1.5.

^$^Analytes excluded from heatmap analysis because they were not detectable in any of the conditions in more than 50% of cases and controls.

**^¶^**Analytes are excluded from analysis because background levels in medium only condition were above the top limit of detection in all or most of the tested samples, or added during culture (IL-7)

^††^IL-17D is excluded from analysis because it is produced by non-hematopoietic cells only.

^1^IL-8 was tested with two different assays: IL-8 had a dynamic range of 0.04-375 pg/ml, all conditions were above the top limit of detection in this assay and data were excluded from analysis. The IL-8 HA assay had a dynamic range of 95.6-43400 pg/ml and data were included in the analysis.

^2^IL-17A was analyzed with two different assays: IL-17A had a dynamic range of 074-3653 pg/ml. The IL-17A genB assay had a dynamic range of 0.413-1950 pg/ml.

**Supplemental table 3, ROC data, part 1**

| **Function** | **Culture Condition** | **Cyto/chemokine** | **AUC** | **p-value ROC** |
| --- | --- | --- | --- | --- |
| Type 1 | No skewing | TNFα | 0.80** | 0.0029 |
|  | Type 1 skewing |  | 0.87** | 0.0002 |
|  | Type 2 Skewing |  | 0.83** | 0.0010 |
|  | Type 17 skewing |  | 0.83** | 0.0011 |
|  | No skewing | TNFβ | 0.86** | 0.0003 |
|  | Type 1 skewing |  | 0.64* | 0.1700 |
|  | Type 2 Skewing |  | **0.90***** | <0.0001 |
|  | Type 17 skewing |  | 0.67 | 0.0960 |
|  | No skewing | IFNγ | 0.81** | 0.0017 |
|  | Type 1 skewing |  |  | Unreliable |
|  | Type 2 Skewing |  | 0.82** | 0.0015 |
|  | Type 17 skewing |  | 0.85** | 0.0004 |
| Type 1/Type 17 | No skewing | GMCSF | **0.93***** | <0.0001 |
|  | Type 1 skewing |  | 0.80** | 0.0030 |
|  | Type 2 Skewing |  | 0.87** | 0.0002 |
|  | Type 17 skewing |  | 0.75* | 0.0124 |
| Type 1/Type 2 | No skewing | IL-2 | 0.82** | 0.0016 |
|  | Type 1 skewing |  | 0.61 | 0.2800 |
|  | Type 2 Skewing |  | **0.90***** | <0.0001 |
|  | Type 17 skewing |  | 0.54 | 0.6800 |
|  | No skewing | IL-10 | 0.84** | 0.0006 |
|  | Type 1 skewing |  | 0.83** | 0.0010 |
|  | Type 2 Skewing |  | 0.85** | 0.0004 |
|  | Type 17 skewing |  | 0.69 | 0.0574 |
| Type 2 | No skewing | IL-3 | 0.77* | 0.0077 |
|  | Type 1 skewing |  | 0.76* | 0.0093 |
|  | Type 2 Skewing |  | 0.71* | 0.0388 |
|  | Type 17 skewing |  | 0.65 | 0.1300 |
|  | No skewing | IL-4 | 0.86** | 0.0003 |
|  | Type 1 skewing |  | 0.73* | 0.0205 |
|  | Type 17 skewing |  | 0.67 | 0.0860 |
|  | No skewing | IL-5 | **0.95***** | <0.0001 |
|  | Type 1 skewing |  | **0.90***** | <0.0001 |
|  | Type 2 Skewing |  | **0.94***** | <0.0001 |
|  | Type 17 skewing |  | 0.86** | 0.0003 |
|  | No skewing | IL-9 | 0.86** | 0.0003 |
|  | Type 1 skewing |  | 0.81** | 0.0019 |
|  | Type 2 Skewing |  | 0.87** | 0.0003 |
|  | Type 17 skewing |  | 0.86** | 0.0003 |
|  | No skewing | IL-13 | 0.68 | 0.0730 |
|  | Type 1 skewing |  | 0.85** | 0.0004 |
|  | Type 2 Skewing |  | **0.90***** | <0.0001 |
|  | Type 17 skewing |  | 0.81** | 0.0022 |

**Supplemental table 3, ROC data, part 2**

| Function | Culture Condition | Cyto/chemokine | AUC | p-value ROC |
| --- | --- | --- | --- | --- |
| Type 17 | No skewing | IL-17A | 0.84** | 0.0007 |
|  | Type 1 skewing |  | 0.73* | 0.0196 |
|  | Type 2 Skewing |  | 0.79* | 0.0039 |
|  | Type 17 skewing |  | 0.81** | 0.0022 |
|  | No skewing | IL-17AGenB | 0.84** | 0.0007 |
|  | Type 1 skewing |  | 0.81** | 0.0022 |
|  | Type 2 Skewing |  | 0.69 | 0.0574 |
|  | Type 17 skewing |  | 0.77* | 0.0073 |
|  | No skewing | IL-17AF | 0.84** | 0.0007 |
|  | Type 1 skewing |  | 0.81** | 0.0022 |
|  | Type 2 Skewing |  | 0.84** | 0.0007 |
|  | Type 17 skewing |  | 0.67 | 0.0956 |
|  | No skewing | IL-21 | 0.69 | 0.0597 |
|  | Type 1 skewing |  | 0.78* | 0.0049 |
|  | Type 2 Skewing |  | 0.55 | 0.6170 |
|  | Type 17 skewing |  | 0.76* | 0.0103 |
| Type 22 | No skewing | IL-22 | **0.93***** | <0.0001 |
|  | Type 1 skewing |  | 0.84** | 0.0008 |
|  | Type 2 Skewing |  | 0.57 | 0.4800 |
|  | Type 17 skewing |  | **0.93***** | <0.0001 |
| T-cells/APC | No skewing | TARC | **0.90***** | <0.0001 |
|  | Type 1 skewing |  | 0.85** | 0.0005 |
|  | Type 2 Skewing |  | 0.87** | 0.0002 |
|  | Type 17 skewing |  | 0.76* | 0.0100 |
|  | No skewing | IL-8 (HA) | 0.57 | 0.5000 |
|  | Type 1 skewing |  | 0.57 | 0.4600 |
|  | Type 2 Skewing |  | 0.72* | 0.0290 |
|  | Type 17 skewing |  | 0.50 | 0.9700 |
| APC | No skewing | IP-10 | 0.79* | 0.0039 |
|  | Type 1 skewing |  | 0.53 | 0.8900 |
|  | Type 2 Skewing |  | 0.87** | 0.0002 |
|  | Type 17 skewing |  | 0.78* | 0.0060 |
|  | No skewing | IL-27 | 0.74* | 0.0160 |
|  | Type 1 skewing |  | 0.58 | 0.4300 |
|  | Type 2 Skewing |  | 0.63 | 0.1900 |
|  | Type 17 skewing |  | 0.85** | 0.0005 |
|  | No skewing | IL-12/23p40 | 0.69 | 0.0550 |
|  | Type 2 skewing |  | 0.78* | 0.0050 |
|  | No skewing | MIP3α | 0.69 | 0.0570 |
|  | Type 1 skewing |  | 0.63 | 0.1900 |
|  | Type 2 Skewing |  | 0.65 | 0.1300 |
|  | Type 17 skewing |  | 0.61 | 0.2700 |

AUC: area under the curve ***Excellent accuracy for diagnostic test: 0.90-1.00; **Good accuracy for diagnostic test: 0.80-0.90; *Fair accuracy for diagnostic test: 0.70-0.80

**Supplemental table 4: Raw data (pg/ml) of top 10 analyte/culture condtition combinations**

Values in red are below of above linear range of the assay, bold values in nickel conditions were considered positive based on SI. N.D. = not detected, PT=nickel patch test, H=clinical history of nickel allergy

**Supplemental table 5: Correlation lymphocyte proliferation and cytokine production tests**

| **Test protocol lymphocyte proliferation** | **Test protocol cytokine production** | **Spearmans ρ** | **P** |
| --- | --- | --- | --- |
| LPT autologous serum – no skewing | *IL-5* – no skewing | 0.88 | < 0.0001 |
|  | *GM-CSF* – no skewing | 0.76 | < 0.0001 |
|  | *IL-22* – no skewing | 0.76 | < 0.0001 |
|  | *TARC* – no skewing | 0.69 | < 0.0001 |
| LPT autologous serum – Type 2 skewing | *IL-5* – Type 2 skewing | 0.81 | < 0.0001 |
|  | *IL-13* – Type 2 skewing | 0.76 | < 0.0001 |
|  | *IL-2* – Type 2 skewing | 0.61 | < 0.0001 |
|  | *TFNβ* – Type 2 skewing | 0.77 | < 0.0001 |

LPT: lymphocyte proliferation test

**Supplemental figure 1 legend:**

Unsupervised cluster analysis: Dark blue colors represent low SI’s, white colors represent average SI’s, and dark red represent high SI’s.

patch test negative, no history of nickel allergy (Controls): ; patch test positive, history of nickel allergy (true positives): ; patch test negative, history of nickel allergy ; patch test positive, no history of nickel allergy: . A: non skewed, B: type 1 skewed, C: type 2 skewed, D: type 17 skewed cultures
