# Supplementary figures and images for: Nickel allergy is associated with a broad spectrum cytokine response
Source: Contact Dermatitis. 2022 Sep 8;88(1):10–7. doi: 10.1111/cod.14199 (PMC10087880; doi:10.1111/cod.14199)

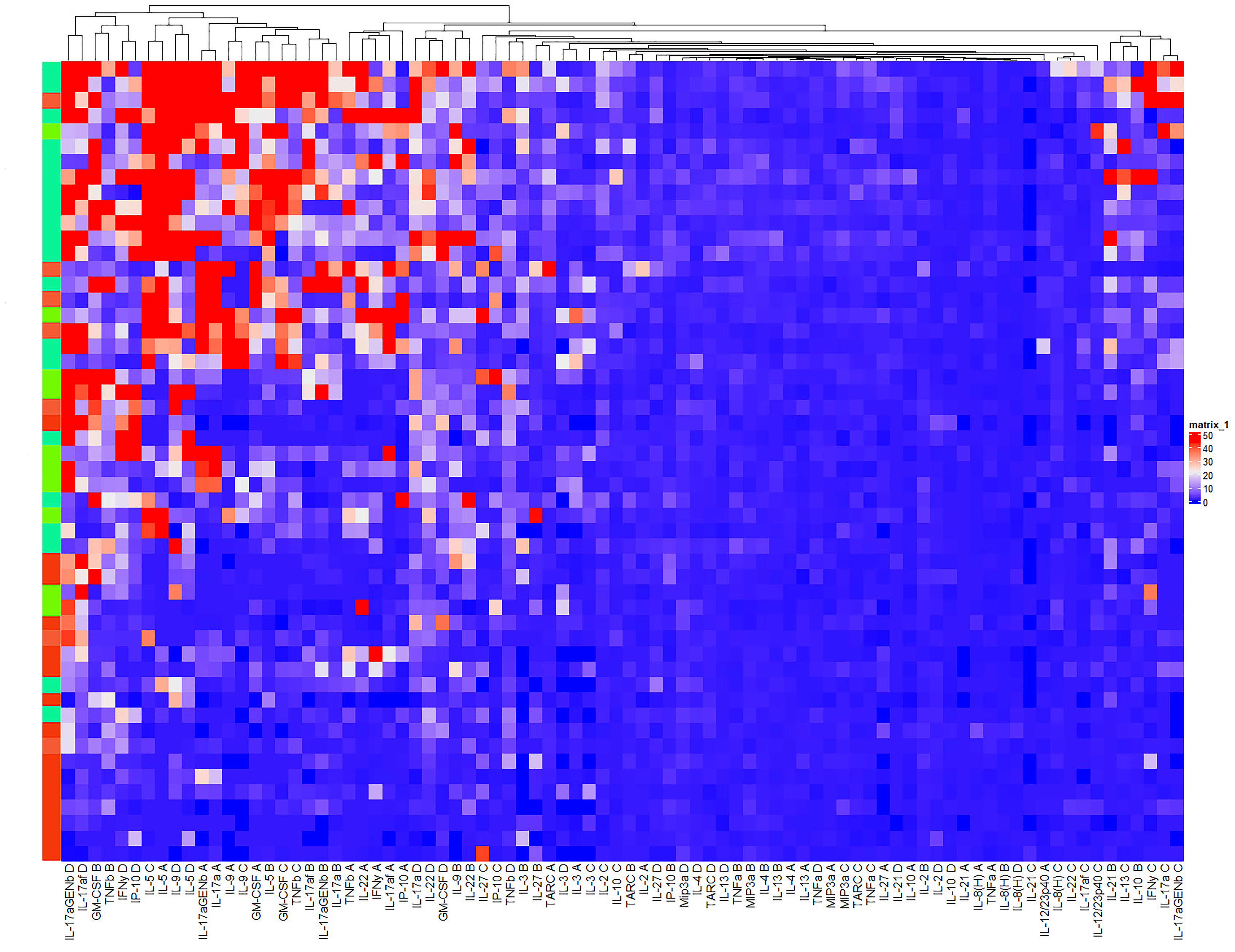

Supplement: Supplementary file 2 — Figure S1 Unsupervised cluster analysis: Dark blue colours represent low SI's, white colours represent average SI's, and dark red represent high SI's. [file COD-88-10-s001.jpg]
